# Vitamin K antagonists: relative strengths and weaknesses vs. direct oral anticoagulants for stroke prevention in patients with atrial fibrillation

**DOI:** 10.1007/s11239-016-1446-0

**Published:** 2016-11-28

**Authors:** Andreas Zirlik, Christoph Bode

**Affiliations:** Department of Cardiology and Angiology I, University Heart Centre Freiburg, Hugstetter Strasse 55, 79106 Freiburg, Germany

**Keywords:** Anticoagulants, Antithrombins, Atrial fibrillation, Factor Xa inhibitors, Stroke

## Abstract

Vitamin K antagonists (VKAs) have been the mainstay of anticoagulation therapy for more than 50 years. VKAs are mainly used for the prevention of stroke in patients with atrial fibrillation (AF) and the treatment and secondary prevention of venous thromboembolism. In the past 5 years, four new agents—the direct factor Xa inhibitors apixaban, edoxaban and rivaroxaban and the direct thrombin inhibitor dabigatran [collectively known as direct oral anticoagulants (DOACs) or non-VKA oral anticoagulants]—have been approved for these and other indications. Despite these new treatment options, the VKA warfarin currently remains the most frequently prescribed oral anticoagulant. The availability of DOACs provides an alternative management option for patients with AF, especially when the treating physician is hesitant to prescribe a VKA owing to associated limitations, such as food and drug interactions, and concerns about bleeding complications. Currently available real-world evidence shows that DOACs have similar or improved effectiveness and safety outcomes compared with warfarin. Treatment decisions on which DOAC is best suited for which patient to maximize safety and effectiveness should take into account not only clinically relevant patient characteristics but also patient preference. This article reviews and highlights real and perceived implications of VKAs for the prevention of stroke in patients with non-valvular AF, with specific reference to their strengths and weaknesses compared with DOACs.

**Electronic supplementary material:**

The online version of this article (doi:10.1007/s11239-016-1446-0) contains supplementary material, which is available to authorized users.

## Introduction

Vitamin K antagonists (VKAs) provide effective anticoagulation and have been the mainstay of anticoagulation therapy for more than 50 years. VKAs are mostly used as long-term anticoagulant therapy, including for the prevention of stroke in patients with atrial fibrillation (AF) and the treatment of venous thromboembolism (VTE). Warfarin currently remains the most frequently prescribed oral anticoagulant (OAC) for these indications, even with the approval within the past 5 years of four new agents, namely the direct factor Xa inhibitors apixaban, edoxaban and rivaroxaban and the direct thrombin inhibitor dabigatran [collectively known as novel/non-VKA/direct OACs (DOACs)].

On average, patients with AF have a five-fold higher risk of stroke than patients without AF—irrespective of whether they have paroxysmal or chronic AF [123]. AF-related strokes are associated with an approximately 50% increased risk of disability and a 60% increased risk of death at 3 months compared with strokes of other aetiologies [83]. The number of strokes caused by AF-related thromboembolisms may be even higher than currently thought because data from recent studies have shown that cryptogenic strokes (i.e. those without a well-defined aetiology) account for approximately 30% of ischaemic strokes [112]. Evidence suggests that up to 30% of patients with cryptogenic stroke may have AF [54, 110]. Therefore, effective anticoagulation is vital for the long-term management of patients with AF at an intermediate or high risk of stroke [27, 125]. VKAs reduce the rate of stroke by approximately 60% [65], whereas antiplatelet therapy is much less efficacious (reducing the event rate by approximately 20%) and has almost the same bleeding risk as oral anticoagulation therapy [2, 85]. Despite guideline recommendations and clear evidence that oral anticoagulation therapy is indicated in patients with AF and who have a CHA_2_DS_2_-VASc score of ≥1, several studies report that, on average, only 60% of eligible patients receive anticoagulation therapy.

The pharmacological characteristics of VKAs, particularly their narrow therapeutic window combined with many drug–drug and drug–food interactions, necessitate regular coagulation monitoring and dose adjustments [3, 5]. An important measure for anticoagulation control with VKAs is the percentage of time a patient spends within the target therapeutic range [i.e. international normalized ratio (INR) of 2.0–3.0]. A number of studies have shown that INR control of VKA therapy is suboptimal in routine clinical practice and, in general, patients spend approximately 40% of time outside the recommended INR range. Poor INR control is associated with increased risk of stroke (INR <2.0) and bleeding (INR >3.0) [88, 122]. Real-life evidence demonstrates that time in therapeutic range (TTR) also varies depending on care setting, such as whether patients are managed by a dedicated anticoagulation clinic or by their general practitioner, cardiologist or neurologist [91].

DOACs have been developed to overcome the limitations of VKA therapy. These agents are now approved in many countries worldwide for the prevention of stroke and systemic embolism in patients with non-valvular AF, as well as for other thromboembolic disorders (Table 1) based on data from phase III, randomized clinical trials [11, 20, 22, 38]. DOACs were at least as good as, if not superior to, warfarin in terms of efficacy for the prevention of stroke in patients with non-valvular AF and also offered a better safety profile (all four major trials consistently indicated reduced rates of intracranial and fatal or life-threatening bleeding compared with the respective warfarin arm) [32, 52, 58, 102, 109]. Real-world evidence of DOACs is accumulating, and available data support the findings of the phase III clinical studies (for example [13, 15, 16, 18, 26, 117, 121]). In general, current guidelines recommend DOACs in preference to VKAs [27, 114] or as an alternative to VKAs [74, 94] for prevention of stroke or systemic embolism in patients with non-valvular AF. However, VKAs are still regarded as the ‘gold standard’ by some physicians and continue to be prescribed to many patients, including those who have had difficulties maintaining their INR within the target therapeutic range [48, 88].

**Table 1:** Indications and dosing regimen of DOACs in the EU [11, 20, 22, 38]

	Factor Xa inhibitor			Direct thrombin inhibitor
	Apixaban^a^	Edoxaban^a^	Rivaroxaban^a^	Dabigatran^b^
Prevention of VTE after elective hip or knee replacement surgery	2.5 mg bid	(Not approved)	10 mg od	220 mg od (as two tablets of 110 mg)^c^
Treatment of DVT/PE and prevention of recurrent DVT/PE	10 mg bid for 7 days followed by 5 mg bid; 2.5 mg bid for prevention of recurrence (following 6 months of treatment)	60 mg od (following parenteral anticoagulant for at least 5 days)^d^	15 mg bid for 3 weeks followed by 20 mg od^e^	150 mg bid (following parenteral anticoagulant for at least 5 days)^f^
Prevention of stroke and systemic embolism in patients with non-valvular AF with ≥1 risk factors	5 mg bid^g^	60 mg od^d^	20 mg od^h^	150 mg bid^f^
Prevention of atherothrombotic events in patients with elevated cardiac biomarkers after an ACS in combination with antiplatelet therapy	(Not approved)	(Not approved)	2.5 mg bid	(Not approved)

*ACS* acute coronary syndrome, *AF* atrial fibrillation, *bid* twice daily, *DOAC* direct oral anticoagulant, *DVT* deep-vein thrombosis, *od* once daily, *PE* pulmonary embolism, *VTE* venous thromboembolism

^a^Not recommended in patients with CrCl <15 mL/min

^b^Contraindicated in patients with CrCl <30 mL/min

^c^Started with a half dose 1–4 h after completion of surgery followed by full doses from the next day onwards; reduced dose of 150 mg od (taken as two tablets of 75 mg) in patients with one or more of the following: CrCl 30–50 mL/min; receiving concomitant verapamil, amiodarone or quinidine; aged ≥75 years

^d^Reduced dose of 30 mg od in patients with non-valvular AF or VTE plus one or more of the following clinical factors: CrCl 15–50 mL/min; low body weight ≤60 kg; concomitant use of the following P-glycoprotein inhibitors: cyclosporine, dronedarone, erythromycin or ketoconazole

^e^After the initial dosing period of 15 mg bid for 3 weeks, a reduced dose of 15 mg od should be considered if the patient’s assessed risk for bleeding outweighs the risk for recurrent VTE

^f^Reduced dose of 110 mg bid in patients with non-valvular AF or VTE aged ≥80 years or receiving concomitant verapamil; consider this reduced dose based on individual assessment of thromboembolic risk and bleeding risk in: patients aged 75–80 years, patients with CrCl 30–49 mL/min; patients with gastritis, oesophagitis or gastroesophageal reflux, and other patients at increased risk of bleeding

^g^Reduced dose of 2.5 mg bid in patients with non-valvular AF and serum creatinine ≥1.5 mg/dL (133 µmol/L) plus age ≥80 years and/or body weight ≤60 kg

^h^Reduced dose of 15 mg od in patients with non-valvular AF and CrCl 15–50 mL/min

This article highlights real and perceived implications of VKAs for the prevention of stroke in patients with non-valvular AF, with specific reference to their strengths and weaknesses compared with DOACs. Furthermore, it provides practical guidance on which patients should be switched from VKA to DOAC therapy, which patients should stay on VKA therapy and which DOAC should be given to which patient. Finally, this paper discusses the most suitable overall approach to reducing the burden of AF-related stroke.

## Characteristics of vitamin K antagonist therapy: why it works and areas of inadequacy

The pharmacological characteristics of different VKAs, such as warfarin, phenprocoumon and acenocoumarol (Table 2) are associated with several advantages and practical limitations [3, 44].

**Table 2:** Overview of pharmacological characteristics of direct oral anticoagulants and vitamin K antagonists [3, 59, 66, 71, 106, 119]

Characteristics	Dabigatran	Apixaban	Rivaroxaban	Edoxaban	Warfarin	Acenocoumarol	Phenprocoumon
Target	Factor II	Factor Xa	Factor Xa	Factor Xa	Factors II, VII, IX and X, protein S and C	Factors II, VII, IX and X, protein S and C	Factors II, VII, IX and X, protein S and C
Oral bioavailability (%)	3–7	50	80–100^a^	62	~100	S-Acc: 60 R-Acc: ~100	~100
t_max_ (h)	0.5–2	1–4	2–4	1–2	1.5	1–4	1–4
Half-life (h)	12–17	8–12	5–13	10–14	S-warfarin: 21–43 R-warfarin: 37–89	S-acenocoumarol: 0.5 R-acenocoumarol: 9.0	S-phenprocoumon: 132 R-phenprocoumon: 132
Protein binding (%)	34–35	87	92–95	55	>99	>98	>99
Renal clearance of absorbed active drug (%)	80	27	33	50	80	65	65
CYP substrate	No	3A4/5	3A4, 2J2	3A4/5	2C9	2C9	2C9
P-gp substrate	Yes	Yes	Yes	Yes	No	No	No
Food interaction	No	No	No^b^	NR	Yes	Yes	Yes
Routine coagulation monitoring required	No	No	No	No	Yes	Yes	Yes

*CYP* cytochrome P450, *NR* not reported, *P-gp* P-glycoprotein, *R*- (R)-enantiomer, *S*- (S)-enantiomer, *t*_*max*_ time to reach maximal plasma concentration

^a^Rivaroxaban 20 mg: 66% under fasting conditions (mean area under the plasma concentration–time curve increased by 39% when given with food)

^b^The 15 and 20 mg doses of rivaroxaban should be taken with food to enhance their absorption

VKAs have several inherent advantageous characteristics. They are not eliminated by the kidneys and, therefore, can be used in patients with severe renal impairment. Moreover, the need for regular INR monitoring encourages regular physician–patient contact despite being inconvenient and imposing additional costs. However, although regular physician visits may be beneficial from a medical point of view, poor medication adherence is usually attributable to multiple, interlinked factors and there is no evidence that regular physician visits alone can increase patient adherence [23].

In the case of a missed VKA dose, patients are at less immediate risk of a thrombotic event than patients missing a dose of DOAC, and non-adherent patients may benefit from the slow offset of action. However, (similar to initiation of therapy) reinitiating therapy after missing several doses of a VKA may actually result in a profound pro-thrombotic state [3, 8]. Many physicians are highly familiar with the management and the responsible use of VKAs. Moreover, drug costs of VKAs are significantly lower than those of DOACs. Therefore, physicians may be hesitant to prescribe any of the DOACs.

**Fig. 1:**
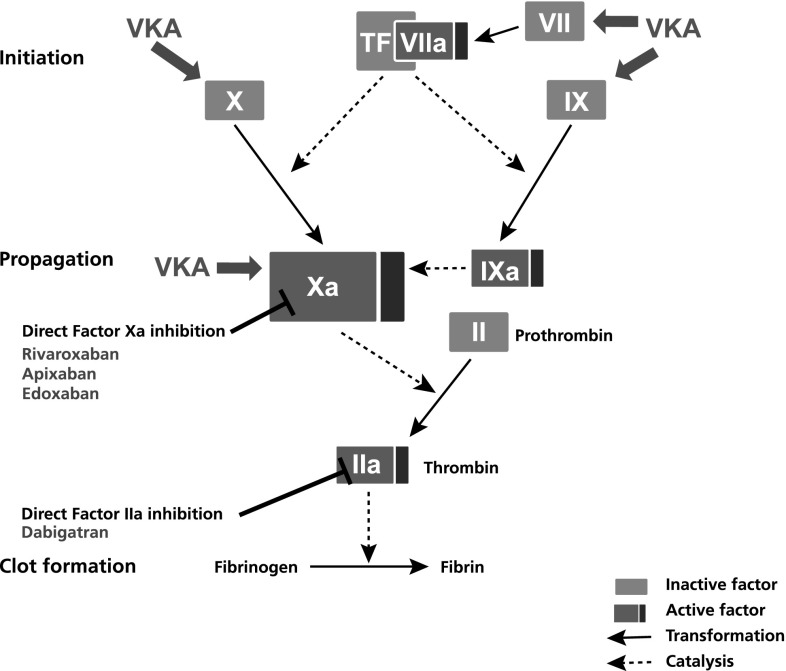
Coagulation cascade with sites of inhibitions for VKAs and direct oral anticoagulants indicated. Coagulation factors are indicated using their factor *numbers in roman numerals, with ‘a’* indicating an active factor. *TF* tissue factor, *VKA* vitamin K antagonist

On the downside, VKAs have an indirect anticoagulant mechanism of action, impairing the synthesis of several vitamin K-dependent coagulation factors (Fig. 1), which results in a slow onset and offset of the anticoagulant effect. On initiation, VKAs are inherently prothrombotic (a fact often overlooked by physicians) because they inhibit the natural anticoagulant proteins C and S faster than inhibiting the coagulation factors X, IX, VII and II: this creates a temporary imbalance in favour of procoagulation factors [3, 8]. In a large case-control study in more than 70,000 patients with AF, warfarin was associated with a 71% increased risk of stroke in the first 30 days of treatment compared with longer periods of treatment [8]. Hence, bridging therapy with a fast-acting, parenteral anticoagulant (e.g. enoxaparin, unfractionated heparin) is necessary on initiation of VKA therapy. For surgery or other interventional procedures, the slow offset of action may delay the procedure. Furthermore, bridging therapy with a fast-acting anticoagulant may also be necessary after the procedure to ensure efficient anticoagulation.

**Fig. 2:**
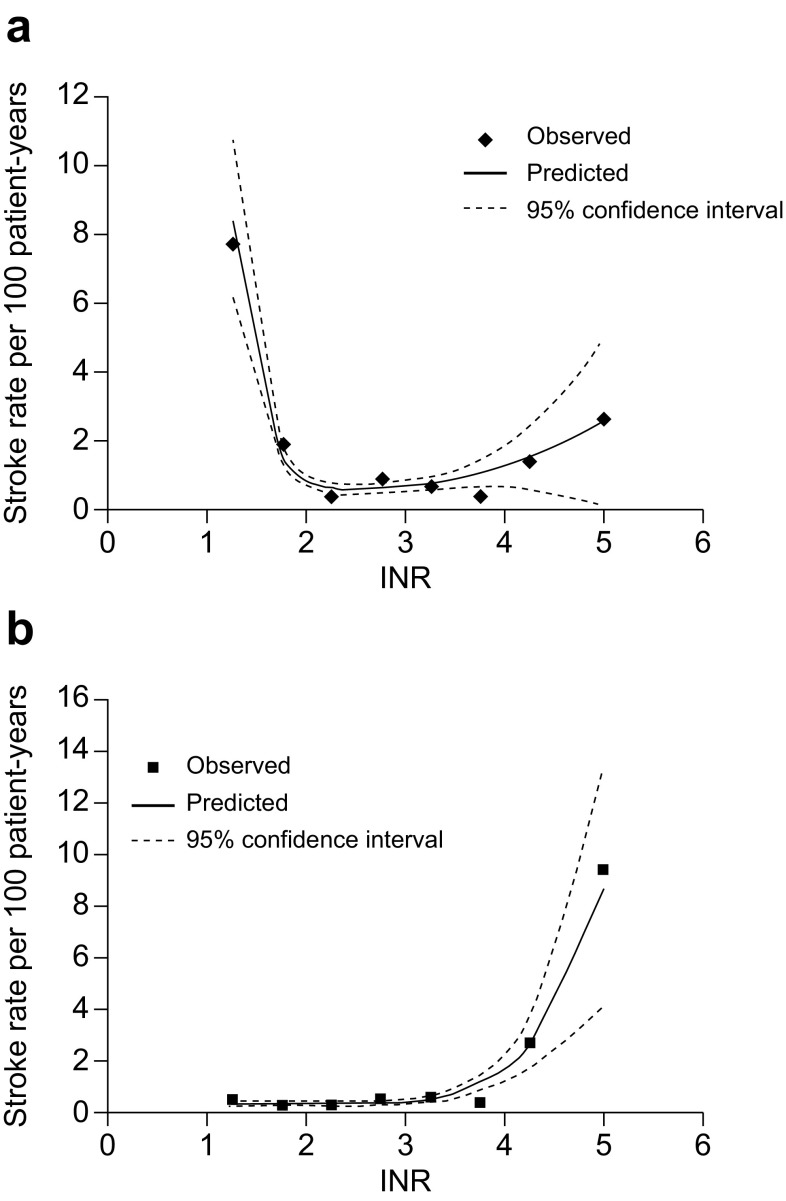
Observed and predicted risk of **a** ischaemic stroke and **b** haemorrhagic stroke according to INR [5]. Reprinted from European Journal of Internal Medicine, Vol 20, Amouyel P, Mismetti P, Langkilde LK, et al. INR variability in atrial fibrillation: A risk model for cerebrovascular events. Pages 63–69, Copyright 2009, with permissions from Elsevier

VKAs have a narrow therapeutic range (Fig. 2) and, therefore, require regular coagulation monitoring and dose adjustments in some patients to keep the anticoagulation intensity within the therapeutic range [3]. Data show that patients on VKAs are effectively anticoagulated only approximately 60% of the time, or even less in some countries [6, 88]. Keeping patients within the target therapeutic range is further complicated by VKAs having multiple food and drugs interactions [3]. These factors can have a significant impact on patients’ daily lives, such as considerable time spent in the clinic for coagulation monitoring and dietary restrictions, all of which may reduce patients’ quality of life [3]. Furthermore, routine anticoagulation monitoring confers additional costs: the cost of the test itself, travel, nurse visits, missed work and the increased workload for physicians and other healthcare staff [1].

## Characteristics of the direct oral anticoagulants: what renders them so attractive and what are their limitations?

The pharmacological characteristics of DOACs provide many practical advantages over VKA therapy (Table 2). Direct targeting of factor Xa or thrombin allows for a much faster effective anticoagulation effect—within 0.5–4 h [51, 62, 89, 92]—and a faster offset of action as opposed to the indirect mode of action via multiple coagulation factors as in the case of VKAs (Fig. 2). Moreover, the kinetics of DOACs (e.g. rivaroxaban [78]) closely mimic those of the low-molecular-weight heparin enoxaparin. Therefore, in contrast to VKAs, bridging with a parenteral anticoagulant is not necessary with the DOACs [66]. DOACs also have a much shorter half-life compared with VKAs, making bridging to interventions or surgery obsolete [67].

DOACs have predictable pharmacokinetics and pharmacodynamics and a lower potential for food and drug interactions [11, 20, 22, 38]. These agents can, therefore, be given at fixed dosing schedules without the need for dietary restrictions or routine coagulation monitoring. However, the lack of the requirement for routine monitoring does not negate the need for regular physician–patient contact and patients should schedule regular visits. Although the frequency of these visits should be determined by bleeding risk (HAS-BLED score), age and renal function, patients are recommended to return every 3 months for a review of their treatment [67]. Measurement of the anticoagulation effect and/or drug levels may be helpful in certain clinical circumstances, such as in the event of suspected overdose, during bleeding events, prior to urgent surgery, in patients with deteriorating renal function or when determining the use of thrombolysis.

Unlike VKAs, DOACs are eliminated renally, albeit at different rates; renal impairment affects exposure and the associated risk of bleeding. Renal clearance of the absorbed active drug is approximately 27% for apixaban [22], 35% for rivaroxaban [92], 50% for edoxaban [38] and >80% for dabigatran [46]. Based on these characteristics, apixaban, edoxaban and rivaroxaban are not recommended in patients with AF and who have creatinine clearance (CrCl) <15 mL/min [11, 22, 38] and dabigatran is contraindicated in patients with CrCl <30 mL/min [20]. Furthermore, edoxaban should be used with caution in patients with high CrCl because of reduced efficacy [38]; In the US, edoxaban should not be used in patients with CrCl >95 mL/min [39].

## Vitamin K antagonists: performance in real-world practice

### Time in therapeutic range and real-world effectiveness and safety

VKAs can only provide clinical benefit if the anticoagulation effect is kept within the therapeutic range (INR 2.0–3.0); poor INR control can lead to an increased risk of thromboembolism (INR <2.0) or bleeding (INR >3.0) [5]. TTR during VKA therapy is higher during controlled clinical studies than in daily practice, owing to their strict study protocols and the regular follow-up with patients. Moreover, TTR control in daily clinical practice is also dependent on whether the patient is managed by a dedicated anticoagulation clinic or elsewhere, such as by a general practitioner, cardiologist or neurologist [91]. The rate of fatal and major bleeding events was low (0.25 and 1.1% per year, respectively) in patients whose anticoagulation with warfarin was managed by an anticoagulation clinic achieving a median TTR of 68% [91, 101].

The inability to maintain TTR is well reported: in the Registry of Canadian Stroke Network, 74% of patients with known AF who were taking warfarin at the time of ischaemic stroke had sub-therapeutic anticoagulation [53]. Additional evidence emphasizes that stroke prevention with a VKA is effective in patients who have a good individual mean TTR (>75%) [91]. Data from the GARFIELD-AF registry indicate that only 29% of VKA-treated patients had good anticoagulation control, defined as a TTR ≥70%, and that heavy alcohol use was associated with poor anticoagulation control (TTR ≤60%) [118]. Patients with poor control had a significantly higher risk of death [hazard ratio 2.87; 95% confidence interval (CI) 1.97–4.19] and stroke/systemic embolism (hazard ratio 1.98, 95% CI 1.13–3.47) than those with a TTR >60% [118].

Data collected outside of anticoagulation clinics (and, therefore, most likely in patients with suboptimal anticoagulation control) suggest that real-world effectiveness fails to reproduce efficacy data for VKAs seen in clinical studies. However, warfarin has been shown to prevent stroke and systemic embolism more effectively than placebo or acetylsalicylic acid. A large meta-analysis of clinical study data demonstrated a 62% reduction in the risk of stroke and systemic embolism with warfarin therapy compared with placebo/acetylsalicylic acid therapy [64]. A real-world Canadian study in patients with AF showed that warfarin-treated outpatients had a significantly lower risk of stroke compared with patients who did not receive any antithrombotic treatment (risk ratio 0.31) [29]. The risk of bleeding is much higher in clinical practice compared with the rates reported in clinical studies. A large cohort study in 125,195 patients with AF demonstrated a high risk of haemorrhage during the first 30 days of warfarin therapy (11.8% per year): considerably higher than the rates of 1–3% reported in randomized controlled trials [55]. In the GARFIELD-AF registry, treatment at an anticoagulation clinic or thrombosis centre was associated with a better TTR compared with other settings (proportion of patients with TTR >60%: 57.1 vs. 46.2%) [118].

There are efforts to simplify the management of VKAs by way of patient self-testing and self-management. Initial clinical trials had encouraging outcomes [19]; however, patients in these trials had a high level of education, which is not necessarily a true representation of all patients encountered in daily clinical practice. Finally, studies trying to optimize the benefit–risk ratio of VKAs by lowering the INR range to 1.5–2.5 failed, with inferior efficacies but similar bleeding compared with standard-dose VKA therapy [103].

### Patient preference and compliance to vitamin K antagonist therapy

Limitations and inconveniences that both physicians and patients associate with VKA therapy are contributing to their under-prescription in patients with high risk of stroke and systemic embolism. In the GARFIELD-AF registry, 38.0% of patients with a CHADS_2_ score ≥2 did not receive anticoagulant therapy; 7.2% of patients with AF and CHADS_2_ ≥2 had refused treatment for various reasons, including inconvenience of regular blood tests, dietary restrictions, bleeding risk and an under-appreciation or lack of knowledge regarding the risk of stroke.

As well as being unwilling to start VKA therapy, many patients with AF who are initiated on VKA therapy discontinue or are non-adherent [47, 56, 77, 98]. For example, of 125,195 patients newly diagnosed with AF in Canada from 1997 to 2008, 9% did not collect their second prescription of warfarin within the first half year and 32% discontinued therapy within 1 year, rising to 43% at 2 years and 61% at 5 years [56]. Similarly, in a US study, more than one in four new warfarin starters discontinued therapy within a year [47]. In another study, 40% of patients were non-adherent to VKA therapy (>20% of days with missed doses or >10% of days where extra doses were taken in addition to the prescribed dose), and this percentage was significantly associated with poor anticoagulation control [77].

### Underuse and inappropriate use of anticoagulation therapies

Large registries published between 2005 and 2009 by the European Heart Survey, the German Competence NETwork on AF (AFNET) and the Canadian Stroke Network suggest that 30–60% of patients with AF who are eligible according to guidelines are not prescribed anticoagulation therapy [53, 93, 97]. In the Registry of Canadian Stroke Network, only 10% of patients with acute stroke with known AF were therapeutically anticoagulated at time of hospital admission [53]. Underuse of anticoagulation in these patients had unfavourable implications: approximately 80% of the resulting strokes were disabling or fatal [53]. The global GARFIELD-AF registry (2009–2011) found that 34% of patients with a CHADS_2_ score ≥2 received antiplatelet therapy [76]. Of patients with a CHADS_2_ score ≥2 who received anticoagulation therapy, 62% received a VKA. In addition, 43% of patients with a CHADS_2_ score of 0 received anticoagulation therapy.

Taken together, there appears to be underuse of anticoagulation therapy in patients at moderate to high risk of stroke and systemic embolism and overuse in patients at low risk—demonstrating that, in real-life practice, prescribed therapy is often not based on evidence-based risk schemes and guidelines [76]. In almost half of the cases (48.3%) in which VKA therapy was not prescribed, this was the physician’s choice and not based on guidelines or contraindications to therapy; the physician’s reasons included concerns over bleeding risk (7.4%), concerns over the risk of falling (6.5%), concerns over patient compliance (5.3%) and perceived low risk of stroke (4.1%). Many of the concerns given as reasons for not prescribing VKA are not supported by actual data. For example, a prospective study in patients on OACs at high risk of falls did not have a significantly increased risk of major bleeding events [40].

Poor TTR in everyday clinical practice, coupled with low rates of adherence or high discontinuation rates, and a general underuse of VKA therapy supports the need for alternative oral anticoagulation options that are easier to manage and more convenient than VKA therapy. In the next sections, we review clinical studies and real-world data, with the practical advantages associated with DOAC therapy compared against the aforementioned limitations associated with VKAs.

## Vitamin K antagonists vs. direct oral anticoagulants: outcomes of phase III studies and real-life evidence

### Efficacy and safety

Results from phase III trials of DOACs, with a wide range of patients with AF worldwide, showed that all DOACs are at least as effective as warfarin, with similar or lower rates of major bleeding [32, 34, 52, 58, 102]. Importantly, a direct, head-to-head comparison of these studies is not feasible because the study designs and study populations were different. In a meta-analysis of all four DOACs in phase III trials for stroke/systemic embolism prevention in patients with AF vs. warfarin, these agents reduced the risk of haemorrhagic stroke by 51% and the risk of intracranial haemorrhage by 52% [109].This favourable benefit–risk profile extends to many subgroups and ethnicities including the Asian population in which the rate of intracranial bleeding is reduced by up to 80% (as reviewed elsewhere [31, 72, 73]). Conversely, the risk of gastrointestinal bleeding was 25% higher with DOACs than with warfarin, owing to bleeding events with dabigatran (150 mg twice daily), edoxaban (60 mg once daily) and rivaroxaban (20 mg) (Tables 3, 4) [32–34, 43, 52, 58, 102, 109].

**Table 3:** Main efficacy and safety results from the phase III clinical trials of the direct oral anticoagulants approved for prevention of stroke in patients with non-valvular atrial fibrillation

	RE-LY [32–34, 43] (Dabigatran vs. warfarin)		ROCKET AF [102] (Rivaroxaban vs. warfarin)	ARISTOTLE [58] (Apixaban vs. warfarin)	ENGAGE AF [52] (Edoxaban vs. warfarin)	
	110 mg	150 mg			30 mg	60 mg
Efficacy outcomes (% per year)						
Stroke or SE^a^	1.54 vs. 1.72^b^	**1.12 vs. 1.72** ^b^	2.1 vs. 2.4	**1.27 vs. 1.60**	1.61 vs. 1.50^c^	**1.18 vs. 1.50** ^c^
All-cause mortality	3.75 vs. 4.13	3.64 vs. 4.13	1.9 vs. 2.2	**3.52 vs. 3.94**	**3.80 vs. 4.35**	3.99 vs. 4.35
Myocardial infarction	0.82 vs. 0.64^b^	0.81 vs. 0.64^b^	0.9 vs. 1.1	0.53 vs. 0.61	0.89 vs. 0.75	0.70 vs. 0.75
Safety outcomes (% per year)						
Major bleeding	**2.92 vs. 3.61** ^b,d^	3.40 vs. 3.61^b,d^	3.6 vs. 3.4	**2.13 vs. 3.09** ^d^	**1.61 vs. 3.43**	**2.75 vs. 3.43**
Fatal bleeding	**0.19 vs. 0.33**	0.23 vs. 0.33	**0.2 vs. 0.5**	NR (34 vs. 55 patients)	**0.13 vs. 0.38**	**0.21 vs. 0.38**
ICH	**0.23 vs. 0.76**	**0.32 vs. 0.76**	**0.5 vs. 0.7**	**0.33 vs. 0.80**	**0.26 vs. 0.85**	**0.39 vs. 0.85**
Major GI bleeding	1.36 vs. 1.25	**1.85 vs. 1.25**	**3.2 vs. 2.2**	0.76 vs. 0.86	**0.82 vs. 1.23**	**1.51 vs. 1.23**
Major or NMCR bleeding	**14.66 vs. 18.23** ^b,e^	**16.45 vs. 18.23** ^b,e^	14.9 vs. 14.5^f^	**4.07 vs. 6.01**	**7.97 vs. 13.02**	**11.10 vs. 13.02**

Values in bold indicate a statistically significant difference between the direct oral anticoagulant and warfarin

*GI* gastrointestinal, *ICH* intracranial haemorrhage, *NR* not reported, *NMCR* non-major clinically relevant, *SE* systemic embolism

^a^Intention-to-treat analysis

^b^Data with additional events as per [34] or [33] or [43]

^c^Primary efficacy endpoint in ENGAGE-AF was time to adjudicated stroke or systemic embolic event

^d^Primary safety outcome in RE-LY and ARISTOTLE

^e^Major or minor bleeding (minor bleeding was any bleeding not considered to be a major bleeding event)

^f^Primary safety outcome in ROCKET AF

**Table 4:** Subgroup analyses from the phase III clinical trials of direct oral anticoagulants for prevention of stroke in patients with non-valvular atrial fibrillation (there are currently no subgroup analyses of ENGAGE AF data available for subgroups specified in the table)

	RE-LY subgroups (Dabigatran vs. warfarin)		ROCKET AF subgroups (Rivaroxaban vs. warfarin)	ARISTOTLE subgroups (Apixaban vs. warfarin)
Elderly patients	≥75 years 110 mg bid; 150 mg bid [43]		≥75 years [60]	≥75 years [61]
Stroke or SE	1.89 vs. 2.14	1.43 vs. 2.14	2.29 vs. 2.85	**1.56 vs. 2.19**
Major bleeding	4.43 vs. 4.37	5.10 vs. 4.37	4.86 vs. 4.40	**3.33 vs. 5.19**
CrCl 30–50 mL/min	[68]		[50]	[69]
Stroke or SE	2.32 vs. 2.70^a^	1.53 vs. 2.70^a^	2.32 vs. 2.77	2.11 vs. 2.67^b^
Major bleeding	5.45 vs. 5.49^a^	5.50 vs. 5.49^a^	4.49 vs. 4.70	3.21 vs. 6.44^b^
Diabetes	[21]		[10]	[45]
Stroke or SE	1.76 vs. 2.35	1.46 vs. 2.35	1.89 vs. 2.33	1.39 vs. 1.86
Major bleeding	3.81 vs. 4.19	4.66 vs. 4.19	3.79 vs. 3.90	3.01 vs. 3.13
HF	[49]		[120]	[87]
Stroke or SE	1.90 vs. 1.92	1.44 vs. 1.92	1.90 vs. 2.09	**0.99 vs. 1.80** [HF-LVSD] 1.51 vs. 1.54 [HF-pEF]
Major bleeding	3.26 vs. 3.90	3.10 vs. 3.90	NR	2.77 vs. 3.41 [HF-LVSD] **1.95 vs. 3.17** [HF-pEF]
Prior MI	[70]		[86]	[9]
Stroke or SE	1.55 vs. 1.93^c^	1.46 vs. 1.93^c^	1.42 vs. 2.35	1.47 vs. 1.55^c^
Major bleeding	3.94 vs. 4.52^c^	4.24 vs. 4.52^c^	4.75 vs. 3.61	**2.39 vs. 3.05** ^c^

Statistically significant values (*p* ≤ 0.05) are given in bold

*CrCl* creatinine clearance, *HF* heart failure, *HF-pEF* heart failure with preserved ejection fraction, *HF-LVSD* heart failure caused by left ventricular systolic dysfunction, *MI* myocardial infarction, *NR* not reported, *SE* systemic embolism

^a^Values are for CrCl <50 mL/min. ^b^Values are for CrCl ≤50 mL/min. ^c^Values are for coronary artery disease defined as documented coronary artery disease, history of MI and/or history of coronary revascularization

There are several studies comparing real-life effectiveness and safety of VKAs with DOACs (mainly dabigatran or rivaroxaban vs. warfarin; data for apixaban are emerging; data for edoxaban are currently lacking; Table S1 in the electronic supplementary material). Published studies to date demonstrate similar or improved effectiveness with DOACs compared with VKAs (Table S1 in the electronic supplementary material). Recent publications showed discrepancies in real-world effectiveness and safety outcomes with DOACs compared with previously published database analyses or compared with phase III clinical trial results (Table 3).

Real-life evidence from the international, non-interventional, observational phase IV XANTUS study demonstrates that rates of stroke and major bleeding were low in patients receiving rivaroxaban [26]. Data from the Dresden NOAC Registry suggest that rates of major bleeding may be lower with rivaroxaban, apixaban and dabigatran therapy compared with VKA therapy [13, 16, 18, 90]. Moreover, these data show that real-life rates of major bleeding with rivaroxaban were similar (Dresden NOAC Registry [16, 102]) or lower (XANTUS [26]) compared with findings from ROCKET AF [16, 102]. Other observational studies mainly demonstrate that rivaroxaban and dabigatran have similar or reduced rates of major bleeding compared with VKAs, and reflect the decreased incidence of intracranial haemorrhage and increased incidence of gastrointestinal bleeding [30].

### Adherence, persistence and discontinuation

Adherence is defined as the extent to which the patient acts in accordance with the prescribed interval and dose of the dosing regimen and can also be defined as the percent of doses taken as prescribed [36]. Persistence measures the duration of drug therapy during which the patient takes medication without exceeding the permissible gap (usually 60 days). Two retrospective US database analyses showed that patients with AF were significantly more persistent with rivaroxaban than with warfarin, reporting patient persistence with warfarin dropping to <70% at 6 months of therapy [80, 95]. A retrospective US database analysis demonstrated that persistence was higher with dabigatran than with warfarin at 6 months (72 vs. 53%) and 1 year (63 vs. 39%) [126]. This study also showed that patients with a low-to-moderate stroke risk (CHADS_2_ <2) or with a higher bleeding risk (HEMORR_2_HAGES >3) were more likely to discontinue treatment than patients with a high stroke risk or lower bleeding risk [126]. When comparing persistence or adherence among DOACs, two retrospective analyses of different US databases demonstrated that use of the once-daily medication rivaroxaban was associated with significantly higher rates of persistence at 1-year follow-up or significantly higher adherence (percentage of patients who had a proportion of days covered ≥80% during their follow-up) than with the use of the twice-daily medication dabigatran [37, 96]. A Danish nationwide cohort study in approximately 3000 patients with non-valvular AF reported that over 75% of patients treated with dabigatran adhered to therapy more than 80% of the time (as measured by proportion of days covered) [57]. Published data on real-life adherence with edoxaban and apixaban are not yet available.

In the phase III studies (across various follow-up periods), discontinuation rates were: significantly lower with apixaban compared with warfarin in ARISTOTLE; similar between rivaroxaban and warfarin in ROCKET AF and between edoxaban and warfarin in ENGAGE-AF; but significantly higher with dabigatran compared with warfarin in RE-LY, mainly owing to dyspepsia [32, 52, 58, 102]. VKA discontinuation rates in real-life practice range from 25 to 38% at 1-year follow-up and are higher than those reported in controlled phase III studies (10–35% over a median follow-up period of 1.8–2.8 years) [13, 32, 52, 58, 95, 102, 109]. In an analysis of data collected from patients with AF in the Dresden NOAC Registry, discontinuation rates with dabigatran (25.8% per year) were similar to those observed with VKAs in daily practice, whereas discontinuation rates with rivaroxaban therapy (13.6% per year) were much lower than those with VKA therapy [12, 15]. Persistence probabilities at 1 year were 53.1, 47.3 and 25.5% with rivaroxaban, dabigatran and VKA, respectively, and adherence with a high medication possession ratio (≥80%) was 61.4% for rivaroxaban and 49.5% for dabigatran [14]. Together, real-life data suggest that, in the long-term, patients receiving DOACs have better protection against stroke or systemic embolism than patients receiving a VKA.

In the US, 33–69% of all medication-related hospital admissions are estimated to be attributable to poor medication adherence, with the resulting costs of non-adherence being approximately $100 billion/year [100, 115]. An analysis of adverse events based on hospital data identified warfarin as a medication that was most commonly implicated in hospitalization of adults aged ≥65 years (33.3%) owing to adverse drug effects [24].

## The true cost of vitamin K antagonist therapy

In addition to treatment effectiveness and safety, cost-effectiveness is another consideration for decision making by healthcare professionals who have several therapy options. VKAs are often perceived to have lower costs; however, although costs for the drug itself are lower when comparing with DOACs, the true cost of VKA treatment needs to take into account the expenses related to the general management of therapy. These include routine coagulation monitoring, adverse clinical outcomes during therapy (such as bleeding and thromboembolic events) and as a result of non-adherence.

In clinical practice, the estimated mean numbers of hospitalization days, outpatient visits and AF-related hospitalizations associated with rivaroxaban are reported to be lower than those associated with warfarin [79, 81, 82]. Similar published real-world evidence is not yet available for apixaban, edoxaban or dabigatran.

A cost-modelling analysis suggests that, based on the expected number of thrombotic or bleeding events avoided with use of DOACs vs. warfarin, medical costs are reduced when DOACs are used instead of warfarin/placebo for the prevention of stroke in patients with non-valvular AF or for the treatment of VTE [4]. However, a model simulation based on the Slovenian healthcare payer perspective using 2014 costs demonstrated that cost-effectiveness of the DOACs vs. warfarin is highly sensitive to warfarin anticoagulation control [75]. With a TTR of 60%, the probability that warfarin was a cost-effective option was unlikely (probability 1%). This percentage rises with increasing TTR: at a TTR of 70%, warfarin was more cost-effective than DOACs in half of the simulations [75].

## Reversal of anticoagulant effect and management of bleeding

There is currently limited clinical experience with specific reversal agents for the DOACs. However, although vitamin K is a direct, effective reversal agent for VKAs, a normal INR is generally only achieved over approximately 24 h, which would not help in the case of clinically important bleeding events such as intracranial haemorrhage [3, 84]. Therefore, coagulation factor concentrates need to be administered in parallel with vitamin K to restore haemostasis quickly [3, 84, 111].

In most clinical situations, the short half-lives of the DOACs obviate the need for reversal, and standard procedures for bleeding management are normally sufficient to control bleeding events [28]. In fact, specific reversal agents for DOACs would be very rarely needed in daily clinical care. In exceptional clinical situations (such as life-threatening bleeding or emergency surgery associated with a high bleeding risk), coagulation factor concentrates such as prothrombin complex concentrate, activated prothrombin complex concentrate or recombinant factor VIIa may be considered [11, 20, 22, 38]. However, there is limited clinical experience with these agents in patients with bleeding events. Haemostatic agents such as prothrombin complex concentrate or recombinant factor VIIa may increase the risk of thromboembolism if they are administered when the plasma concentration of the anticoagulant is low [124]. Therefore, the risk with the use of these agents has to be balanced with their potential for bleeding control. Recent months have seen the clinical approval of idarucizumab, a specific reversal agent for dabigatran, based on results of a phase III study [104, 105] (Table 5). Moreover, results with a specific reversal agent, andexanet alfa (http://www.clinicaltrials.gov, NCT02220725 and NCT02329327) for factor Xa inhibitors have shown that it has the potential to quickly and effectively reverse the anticoagulation effect of rivaroxaban and apixaban [113]. Andexanet alfa is expected to be approved in 2017 [107]. PER977 (Perosphere) is being assessed as a reversal agent for edoxaban in clinical trials with promising preliminary results [7, 35] (http://www.clinicaltrials.gov, NCT02207257).

**Table 5:** Reversal agents for DOACs

DOAC	Reversal agent, description	Approval status	References
Dabigatran	Idarucizumab (Praxbind^®^): a fully humanized, monoclonal antibody fragment designed to specifically reverse the anticoagulant effect of dabigatran	FDA and EMA	[25, 104, 105]
Factor Xa inhibitors (rivaroxaban and apixaban tested)	Andexanet alfa: an inactive, recombinant version of the human factor Xa designed to specifically reverse the anticoagulant effect of factor Xa inhibitors	Anticipated approval in 2016	[113]
All DOACs, UFH and LMWH	PER977: a small, synthetic, water-soluble, cationic molecule that is designed to bind specifically to UFH and LMWH through non-covalent hydrogen bonding and charge–charge interactions and similarly also binds to edoxaban, rivaroxaban, apixaban and dabigatran	In development	[7, 35]

*DOAC* direct oral anticoagulant, *EMA* European Medicines Agency, *FDA* US Food and Drug Administration, *LMWH* low-molecular-weight heparin, *UFH* unfractionated heparin

## When to switch and when not to switch from vitamin K antagonists to direct oral anticoagulants

Patients who have been initiated on VKA therapy can be switched to a DOAC (see individual Summary of Product Characteristics for further details [11, 20, 22, 38]). This switch should be based on a clinical benefit–risk assessment. Specific reasons for switching may include—but are not limited to—poor INR control, stroke/systemic embolism or serious bleeding during VKA therapy, poor compliance (e.g. relating to the inconveniences of VKA therapy), patient preference to switch to a DOAC therapy, reduced long-term costs and fear of bleeding (particularly within the fragile patient population). Switching strategies are reviewed in the updated practical guide of the European Heart Rhythm Association (EHRA) [66].

The effectiveness and safety of switching patients with AF from a VKA to DOAC therapy has been demonstrated in the Dresden NOAC Registry [17, 90]. Data from this registry regarding patients who switched from a VKA to rivaroxaban or dabigatran for stroke prevention or VTE treatment suggest that the potential for bleeding should be monitored carefully in the first few days after the transition, during which residual VKA activity may remain [90]. One study reported that only 75% of VKA patients had an INR measurement documented before they were started on a DOAC; on average, DOAC was started within 2–5 days after the last intake of VKA. At the 30-day follow-up, the rates of major cardiovascular events (0.8%; 95% CI 0.3–1.8) and major bleeding complications (0.3%; 95% CI 0.0–1.0) were low, with a rate of any bleeding of 12.2% (95% CI 9.8–14.8) in patients with and without INR testing of the residual VKA effect [17]. A Danish analysis demonstrated the importance of adherence to the switching protocols outlined in the Summary of Product Characteristics for dabigatran [116]. This study evaluated real-world outcomes in patients with AF: there was an increased risk of thromboembolism and bleeding with dabigatran in previous VKA users. The authors of this study cautiously interpreted these unexpected results as reflecting patient selection and drug-switching practices. Dabigatran use in VKA-naïve patients was reported to be safe [116]. The EHRA practical guide provides a schematic overview of switching protocols from a VKA to a DOAC and vice versa and also emphasizes the importance of adherence to the established switching strategies [66].

Some patients, especially those with good INR control and TTR, may prefer to continue with VKA therapy instead of switching to a DOAC. Patients may also benefit from continued VKA therapy, including those with contraindications to DOAC therapy. For example, patients with end-stage kidney disease (CrCl <15 mL/min) have significantly increased risks of stroke and bleeding compared with patients with normal kidney function [99]. End-stage kidney disease is also associated with reduced activity of cytochrome P450 2C9, leading to lower warfarin dosing requirements [41]. Patients with valvular AF as opposed to non-valvular AF (particularly in those with mechanical valves) should be treated with a VKA because DOACs are not approved in these patients [11, 20, 22, 38]. Moreover, the outcomes of the RE-ALIGN trial that assessed dabigatran vs. warfarin in patients with mechanical valves reinforced the recommendations of the current guidelines against the use of DOACs in these patients. This trial had to be terminated prematurely owing to an excess of thromboembolic and bleeding events among patients receiving dabigatran (150, 220 or 300 mg twice daily) [42].

## Which direct oral anticoagulant for which patient?

In the absence of a head-to-head trial with DOACs (no such trial is planned or ongoing), no direct answer can be provided to this question. The overall aim should be that all patients with AF who are indicated for anticoagulation should receive appropriate therapy. As discussed at the beginning of this article, a substantial proportion of patients with AF who should be receiving anticoagulation are not receiving OAC therapy of any form. DOACs—overcoming several of the limitations of VKAs—offer alternative and potentially preferred therapy options [27] both in treatment-naïve patients with newly diagnosed AF in need of anticoagulation therapy and in patients with AF at risk of stroke and systemic embolism who are not receiving appropriate therapy or who have poorly controlled VKA therapy.

The choice of which DOAC is the right agent for which patient, initially choosing between a direct thrombin inhibitor and factor Xa inhibitor, should be based on the pharmacokinetics/pharmacodynamics and integration of the clinical data with respect to the patient’s characteristics. The following recommendations, based on the EHRA practical guide, can be used for decision making [66]. In patients with renal impairment, factor Xa inhibitors (rivaroxaban, apixaban or edoxaban) should be preferred over dabigatran. Similarly, factor Xa inhibitors have demonstrated no change to the benefit–risk profile in elderly patients and in patients with a pronounced cardiovascular co-morbidity compared with other patient groups (with especially favourable data for rivaroxaban [60, 63, 86]). Patients with a history or high risk of gastrointestinal bleeding may have a lower risk of bleeding complications with apixaban and low-dose edoxaban than with dabigatran, rivaroxaban or high-dose edoxaban; however, dabigatran, rivaroxaban and warfarin may have similar rates of gastrointestinal bleeding in real-life clinical practice [30]. Furthermore, there is some evidence that patients with a high risk for ischaemic stroke may benefit from a direct thrombin inhibitor (i.e. dabigatran) [108]. More data from real-life studies will shed light on which agent provides the best benefit–risk ratio for which patient.

## Conclusions

The availability of DOACs provides an alternative management option for patients with AF, especially when the treating physician is hesitant to prescribe a VKA owing to the associated limitations, such as routine coagulation monitoring and dose adjustments, food and drug interactions and concerns about bleeding complications. Overall, currently available real-world evidence shows that DOACs have similar or improved effectiveness and safety outcomes compared with warfarin. With regards to which DOAC is best suited for which patient to maximize safety and effectiveness, more prospective real-world data are required because database studies show divergent outcomes. Overall, recommendations in the EHRA practical guide suggest actions taking into account not only clinically relevant patient characteristics but also patient preferences. Adherence to therapy is an important factor to achieve best outcomes, and there is some evidence that patients adhere better to once-daily medications compared with those taken twice-daily.

## Electronic supplementary material

Below is the link to the electronic supplementary material.

Supplementary material 1 (DOCX 69 KB)
