# Supplementary material for: Vitamin K antagonists: relative strengths and weaknesses vs. direct oral anticoagulants for stroke prevention in patients with atrial fibrillation
Source: J Thromb Thrombolysis. 2016 Nov 28;43(3):365–79. doi: 10.1007/s11239-016-1446-0 (PMC5337242; doi:10.1007/s11239-016-1446-0)
Supplement: Supplementary file 1 — Supplementary material 1 (DOCX 69 KB) [file 11239_2016_1446_MOESM1_ESM.docx]

**Electronic supplementary material for:**

Vitamin K antagonists – relative strengths and weaknesses versus direct oral anticoagulants for stroke prevention in patients with atrial fibrillation

Andreas Zirlik and Christoph Bode

Department of Cardiology and Angiology, University Heart Centre Freiburg, Freiburg, Germany

Journal of Thrombosis and Thrombolysis

**Table S1: Overview of real-world comparative effectiveness and safety data with oral anticoagulants in patients with atrial fibrillation (PubMed search Jan 2013–Feb 2016: Criteria: >500 patients, primary paper, English language).**

| **Short reference** | **OAC** | **Prospective or retrospective** | **Study type** | **Patients, n** | **Country or region** | **Time period^a^** | **Effectiveness outcome (events/100 patient-years unless specified)** | **Safety outcomes (%/year unless otherwise specified)** |
| --- | --- | --- | --- | --- | --- | --- | --- | --- |
| **Abraham NS, 2015, BMJ [1]** | R, D, W | Retrospective | Database | 25,830 | US | Nov 2010–Sep 2013 | – | GI bleeding events: D=2.29 (W=2.87), R=2.84 (W=3.06) |
| **Al-Khalili F, 2016, Curr Med Res Opin [2]** | R, D, A | Retrospective | Single-centre study | 766 | Sweden | Dec 2011–May 2014 | – | Major bleeding: R=3.6, D=0.9, A=0.9 |
| **Arihiro S, 2016, Int J Stroke [3]** | R, D, A, W | Prospective | Follow-up assessment/ survey | 1,137 | Japan | Sep 2011–Mar 2014 | Stroke/SE: W=3.06%/3 months, DOACs=2.84%/3 months | Major bleeding: W=2.61%/3 months, DOACs=1.11%/3 months |
| **Arshad A, 2014, Pacing Clin Electrophysiol [4]** | D, W, W with heparin bridging | Retrospective | Database | 882 | US | Oct 2010–Oct 2012 | – | Major complications: W=4.3%, D=0.8%, bridged=2.6% |
| **Avgil-Tsadok M, 2016, Thromb Haemost [5]** | D, W | Retrospective | Database | 63,110 | Canada | Jan 1999–Mar 2013 | Stroke: W=2.43 (men), W=2.95 (women), D110=2.83 (men), D110=3.05 (women), D150=1.74 (men), D150=1.76 (women) | Bleeding events: W=8.43 (men), W=6.56 (women), D110=7.47 (men), D110=6.38 (women), D150=4.54 (men), D150=4.40 (women) |
| **Bassiouny M, 2013, Circ Arrhythm Electrophysiol [6]** | D, W | Prospective | Registry | 999 | US | Dec 2010– Jul 2012 | – | Major bleeding: D= 1.1, W=1.6 |
| **Beyer-Westendorf J, 2014, Blood [7]** | R | Prospective | Registry | 1,775 | Germany | Oct 2011–Dec 2013 | – | Major bleeding: R=6.1 |
| **Beyer-Westendorf J, 2014, Br J Clin Pharmacol [8]** | R, D, VKA | Prospective | Registry | 716 | Germany | Oct 2011–Jun 2013 | Major CV events at Day 30 after switching: DOAC=0.8% | Major bleeding at Day 30 after switching: DOAC=0.3% |
| **Bouillon K, 2015, Lancet Haematol [9]** | R, D, VKA (fluindione, W, or acenocoumarol) | Retrospective | Database | 17,410 | France | Jan 2011–Nov 2012 |  | Risk of bleeding for switchers from W to DOAC: 1%, for non-switchers: 2% |
| **Camm AJ, 2016, Eur Heart J [10]** | R | Prospective | Observational | 6,784 | Europe, Israel, Canada | Jun 2012–Dec 2013 | Stroke: R=0.7 | Major bleeding: R=2.1 |
| **Chan KE, 2015, Circulation [11]** | R, D, W | Prospective | Database | 29,977 | US | Oct 2010–Oct 2014 | – | Total major bleeding events/100 patient years: W=47.1, ASA=35.9, D=83.1, R=68.4 |
| **Chan PH, 2016, Heart Rhythm [12]** | D, W | Retrospective | Database (single-centre) | 571 | China | 2010–2013 | Ischaemic stroke: W=6.9%/year, D=1.4%/year |  |
| **Chan YH, 2016, Stroke [13]** | D, VKA | Retrospective | Database | 19,853 | Taiwan | Jun 2012–Dec 2013 | Ischaemic stroke: W=4.73, D=2.96 | GI bleeding (major): W=0.65, D=0.67; Major hospitalized bleeding: W=2.47, D=1.48 |
| **Chang HY, 2015, BMJ [14]** | R, D, W | Retrospective | Database | 46,163 | US | Oct 2010–Mar 2012 | – | D=9.01, R=3.41, W=7.02 |
| **Coleman CI, 2016, Int J Cardiol [15]** | R, W | Retrospective | Database | 5,108 | Germany | Jan 2012–Oct 2013 | Composite endpoint: R=1.97, W=3.68 | – |
| **Dillier R, 2014, Circ Arrhythm Electrophysiol [16]** | R, VKA | Retrospective | Database | 544 | Germany | Feb 2012– May 2013 | – | Major bleeding complications: R=0.4%, VKA=0.4% |
| **Engelberger RP, 2015, Eur J Intern Med [17]** | R | Prospective | – | 537 | Switzerland | Jan 2012–May 2013 | Stroke/SE: R=0.71 | Major bleeding: R=1.41 |
| **Graham DJ, 2015, Circulation [18]** | D, W | Retrospective | Database | 134,414 | US | Oct 2010–Dec 2012 | Ischaemic stroke: W=1.39, D=1.13 | Major bleeding: W=4.39, D=4.27 |
| **Hernandez I, 2015, JAMA Intern Med [19]** | D, W | – | Database | 9,404 | US | Oct 2010–Oct 2011 | – | W=5.9%, D=9.0% |
| **Ho CW, 2015, Stroke [20]** | D, W, ASA | Prospective | Hospital-based AF registry | 8,754 | Hong Kong | Jul 1997– Dec 2011 | Ischaemic stroke: D=2.24 | – |
| **Johansson AK, 2015, Europace [21]** | D | Retrospective | Clinical records from eight centres | 631 | Sweden | Feb 2012–April 2014 | Thromboembolic events=0.53% | – |
| **Kochhäuser S, 2014, Can J Cardiol [22]** | R, D, W | Retrospective | Single-centre study | 1,581 | Canada | Oct 2010–Oct 2013 | Stroke <30 days: 0% for all groups | Clinically important bleeding <30 days: VKA=0.51%, D=0.39%, R=0.35% |
| **Korenstra J, 2016, Europace [23]** | D, acenocoumarol | Retrospective | Single-centre study | 920 | Netherlands | 2010–2013 | Stoke/SE: D=0.8%/year, acenocoumarol=1.0%/year | Major bleeding: D=2.1%/year, acenocoumarol=4.3%/year |
| **Lakkireddy D, 2014, J Am Coll Cardiol [24]** | R, W | Prospective | Registry | 642 | North America | Jan 2012–Mar 2013 | – | Major bleeding: R=1.6%, W=1.9% |
| **Laliberté F, 2014, Curr Med Res Opin [25]** | R, W | Retrospective | Database | 18,270 | US | May 2011–Jul 2012 | Stroke/SE: R=4.6, W=5.9 | Major bleeding: R=3.3, W=2.9 |
| **Larsen TB, 2014, Am J Med [27]** | D, W | Retrospective | Database | 2,398 | Denmark | Aug 2011–May 2013 | Stroke: VKA-naïve (W=3.5, D110=3.6, D150=1.9); VKA-experienced (W=1.4, D110=4.0, D150=2.1) | – |
| **Larsen TB, 2013, J Am Coll Cardiol [28]** | D, W | Prospective | Nationwide cohort study | 13,914 | Denmark | Aug 2011–Dec 2012 | Stroke: W (D150 matched)=3.0, D150=3.5, W (D110 matched)=3.6, D110=2.7 | Major bleeding: W (D150 matched)=2.9, D150=2.2, W(D110 matched)=3.5, D110=2.8 |
| **Larsen TB, 2014, Am J Med [26]** | D, W | Retrospective | Database | 11,315 | Denmark | Aug 2009–May 2013 | – | Major bleeding: VKA-naïve (W=4, D110=4.4, D150=2.3); VKA-experienced (W=2.8, D110=3.6, D150=1.8) |
| **Lauffenburger JC, 2015, J Am Heart Assoc [29]** | D, W | Retrospective | Database | 64,935 | US | Oct 2010–Dec 2012 | Composite endpoint: W=48.6, D=30.2 (/1,000 patient-years) | GI bleeding: W=32.1, D=21.8 (/1,000 patient-years) |
| **Lauffenburger JC, 2015, Pharmacotherapy [30]** | D | Retrospective | Database | 21,033 | US | Oct 2010–Dec 2012 | – | GI bleeding: D=2.1% over follow-up period |
| **Maura G, 2015, Circulation [31]** | R, D, fluindione, W | Retrospective | Database | 104,257 | France | 2011–2012 | Ischaemic stroke or SE: D=2, R=1.4 | Bleeding events: D=3.3, R=4.9 |
| **Nishtala PS, 2016, Int J Cardiol [32]** | D, W | Retrospective | Database | 8,770 | New Zealand | Jul 2011– Dec 2012 | – | GI bleeding events: W=3.35%, D=3.70% (per person-year) |
| **O'Brien EC, 2015, Eur Heart J [33]** | R, W | Prospective | Registry | 7,411 | US | 2010–2012 | – | Major bleeding OAC=4.0 |
| **Ogawa S, 2014, J Stroke Cerebrovasc Dis [34]** | R | Prospective | Registry | 10,038 | Japan | Apr 2012–Dec 2013 | Composite efficacy endpoint: R=0.6% | Major bleeding: R=0.5% |
| **Seeger JD, 2015, Thromb Haemost [35]** | D, W | Prospective | Database | 38,378 | US | Oct 2010–Dec 2012 | Stroke: D=0.77, W=1.07 | Major bleeding: D=4.42, W=6.17 |
| **Sørensen R, 2013, BMJ Open [36]** | D, VKA | Retrospective | Database | 50,754 | Denmark | Jan 1995–Dec 2011 | Thromboembolic event: W=0.3, D110=0.9, D150=1.4 | Bleeding: W=1.5, D110=3.9, D150=1.4 |
| **Steinberg BA, 2013, Circulation [37]** | Not specified | Prospective | Registry | 7,347 | US | Jun 2010–Aug 2011 | – | Major bleeding: OAC alone=1.8%, OAC+ASA=3.0% |
| **Tamayo S, 2015, Clin Cardiol [38]** | R | Retrospective | Database | 27 467 | US | Jan 2013–Mar 2014 | – | Major bleeding: R=2.86 |
| **Vaughan Sarrazin MS, 2014, Am J Med [39]** | D, W | Retrospective | Database | 85,344 | US | 2010–2012 | – | GI bleeding events per 52 person-weeks: D=0.093, W=0.056 |
| **Villines TC, 2015, Thromb Haemost [40]** | D, W | Retrospective | Database | 25,586 | US | Oct 2009– Jul 2013 | Stroke: D=0.92, W=1.32 | Major bleeding: D=3.08, W=3.70 |
| **Yamaji H, 2013, Clin Drug Investig [41]** | D, W (interrupted, bridged by heparin), W (continuous) | Retrospective | Single-centre study | 503 | Japan | Mar 2009–Dec 2011 | Stroke: W=0%, D=0% | Major bleeding complications: W=1%, D=0% |
| **Yao X, 2016, J Am Heart Assoc [42]** | R, D, A, W | Retrospective | Database | 64,661 | US | Nov 2010–Dec 2014 | Stroke/SE: all OACs=1.32 | Major bleeding: all OACs=3.72 |
| **Yap LB, 2014, J Thromb Thrombolysis [44]** | D | Retrospective | Retrospective cohort registry (hospital database) | 510 | Malaysia | 2010–2013 | – | Major bleeding: D=0.4% |
| **Yap LB, 2015, Clin Appl Thromb Hemost [43]** | D, W | Retrospective | Single-centre study records | 1,000 | Malaysia | 2009–2013 | – | Major bleeding: D=0.7, W=0.41 |

A, apixaban; ASA, acetylsalicylic acid; CV, cardiovascular; D, dabigatran; D110, dabigatran 110 mg; D150, dabigatran 150 mg; DOAC, direct oral anticoagulant; GI, gastrointestinal; OAC, oral anticoagulant; R, rivaroxaban; SE, systemic embolism; VKA, vitamin K antagonist; W, warfarin.

^a^Time period for enrolment or for data available in database studies.

References

1. Abraham NS, Singh S, Alexander GC et al (2015) Comparative risk of gastrointestinal bleeding with dabigatran, rivaroxaban, and warfarin: population based cohort study. BMJ 350:h1857

2. Al Khalili F, Lindstrom C, Benson L (2016) The safety and persistence of non-vitamin-K-antagonist oral anticoagulants in atrial fibrillation patients treated in a well structured atrial fibrillation clinic. Curr Med Res Opin 32:779–785

3. Arihiro S, Todo K, Koga M et al (2016) Three-month risk-benefit profile of anticoagulation after stroke with atrial fibrillation: The SAMURAI-Nonvalvular Atrial Fibrillation (NVAF) study. Int J Stroke 11:565–574

4. Arshad A, Johnson CK, Mittal S et al (2014) Comparative safety of periablation anticoagulation strategies for atrial fibrillation: data from a large multicenter study. Pacing Clin Electrophysiol 37:665–673

5. Avgil-Tsadok M, Jackevicius CA, Essebag V et al (2016) Dabigatran use in elderly patients with atrial fibrillation. Thromb Haemost 115:152–160

6. Bassiouny M, Saliba W, Rickard J et al (2013) Use of dabigatran for periprocedural anticoagulation in patients undergoing catheter ablation for atrial fibrillation. Circ Arrhythm Electrophysiol 6:460–466

7. Beyer-Westendorf J, Förster K, Pannach S et al (2014) Rates, management, and outcome of rivaroxaban bleeding in daily care: results from the Dresden NOAC Registry. Blood 124:955–962

8. Beyer-Westendorf J, Gelbricht V, Forster K et al (2014) Safety of switching from vitamin-K antagonists to dabigatran or rivaroxaban in daily care - results from the Dresden NOAC registry. Br J Clin Pharmacol 78:908–917

9. Bouillon K, Bertrand M, Maura G, Blotiere PO, Ricordeau P, Zureik M (2015) Risk of bleeding and arterial thromboembolism in patients with non-valvular atrial fibrillation either maintained on a vitamin K antagonist or switched to a non-vitamin K-antagonist oral anticoagulant: a retrospective, matched-cohort study. Lancet Haematol 2:e150–e159

10. Camm AJ, Amarenco P, Haas S et al (2016) XANTUS: a real-world, prospective, observational study of patients treated with rivaroxaban for stroke prevention in atrial fibrillation. Eur Heart J 37:1145–1153

11. Chan KE, Edelman ER, Wenger JB, Thadhani RI, Maddux FW (2015) Dabigatran and rivaroxaban use in atrial fibrillation patients on hemodialysis. Circulation 131:972–979

12. Chan PH, Huang D, Hai JJ et al (2016) Stroke prevention using dabigatran in elderly Chinese patients with atrial fibrillation. Heart Rhythm 13:366–373

13. Chan YH, Yen KC, See LC et al (2016) Cardiovascular, bleeding, and mortality risks of dabigatran in Asians with nonvalvular atrial fibrillation. Stroke 47:441–449

14. Chang HY, Zhou M, Tang W, Alexander GC, Singh S (2015) Risk of gastrointestinal bleeding associated with oral anticoagulants: population based retrospective cohort study. BMJ 350:h1585

15. Coleman CI, Antz M, Ehlken B, Evers T (2016) REal-LIfe Evidence of stroke prevention in patients with atrial Fibrillation - The RELIEF study. Int J Cardiol 203:882–884

16. Dillier R, Ammar S, Hessling G et al (2014) Safety of continuous periprocedural rivaroxaban for patients undergoing left atrial catheter ablation procedures. Circ Arrhythm Electrophysiol 7:576–582

17. Engelberger RP, Noll G, Schmidt D et al (2015) Initiation of rivaroxaban in patients with nonvalvular atrial fibrillation at the primary care level: the Swiss Therapy in Atrial Fibrillation for the Regulation of Coagulation (STAR) Study. Eur J Intern Med 26:508–514

18. Graham DJ, Reichman ME, Wernecke M et al (2015) Cardiovascular, bleeding, and mortality risks in elderly Medicare patients treated with dabigatran or warfarin for non-valvular atrial fibrillation. Circulation 131:157–164

19. Hernandez I, Baik SH, Piñera A, Zhang Y (2015) Risk of bleeding with dabigatran in atrial fibrillation. JAMA Intern Med 175:18–24

20. Ho CW, Ho MH, Chan PH et al (2015) Ischemic stroke and intracranial hemorrhage with aspirin, dabigatran, and warfarin: impact of quality of anticoagulation control. Stroke 46:23–30

21. Johansson AK, Juhlin T, Engdahl J et al (2015) Is one month treatment with dabigatran before cardioversion of atrial fibrillation sufficient to prevent thromboembolism? Europace 17:1514–1517

22. Kochhauser S, Khaykin Y, Beardsall J et al (2014) Comparison of outcomes after cardioversion or atrial fibrillation ablation in patients with differing periprocedural anticoagulation regimens. Can J Cardiol 30:1541–1546

23. Korenstra J, Wijtvliet EP, Veeger NJ et al (2016) Effectiveness and safety of dabigatran versus acenocoumarol in 'real-world' patients with atrial fibrillation. Europace 18:1319–1327

24. Lakkireddy D, Reddy YM, Di Biase L et al (2014) Feasibility and safety of uninterrupted rivaroxaban for periprocedural anticoagulation in patients undergoing radiofrequency ablation for atrial fibrillation: results from a multicenter prospective registry. J Am Coll Cardiol 63:982–988

25. Laliberté F, Cloutier M, Nelson WW et al (2014) Real-world comparative effectiveness and safety of rivaroxaban and warfarin in nonvalvular atrial fibrillation patients. Curr Med Res Opin 30:1317–1325

26. Larsen TB, Gorst-Rasmussen A, Rasmussen LH, Skjoth F, Rosenzweig M, Lip GYH (2014) Bleeding events among new starters and switchers to dabigatran compared with warfarin in atrial fibrillation. Am J Med 127:650–656

27. Larsen TB, Rasmussen LH, Gorst-Rasmussen A, Skjoth F, Lane DA, Lip GYH (2014) Dabigatran and warfarin for secondary prevention of stroke in atrial fibrillation patients: a nationwide cohort study. Am J Med 127:1172–1178

28. Larsen TB, Rasmussen LH, Skjøth F et al (2013) Efficacy and safety of dabigatran etexilate and warfarin in "real-world" patients with atrial fibrillation: a prospective nationwide cohort study. J Am Coll Cardiol 61:2264–2273

29. Lauffenburger JC, Farley JF, Gehi AK, Rhoney DH, Brookhart MA, Fang G (2015) Effectiveness and safety of dabigatran and warfarin in real-world US patients with non-valvular atrial fibrillation: a retrospective cohort study. J Am Heart Assoc 4:e001798

30. Lauffenburger JC, Rhoney DH, Farley JF, Gehi AK, Fang G (2015) Predictors of gastrointestinal bleeding among patients with atrial fibrillation after initiating dabigatran therapy. Pharmacotherapy 35:560–568

31. Maura G, Blotiere PO, Bouillon K et al (2015) Comparison of the short-term risk of bleeding and arterial thromboembolic events in nonvalvular atrial fibrillation patients newly treated with dabigatran or rivaroxaban versus vitamin K antagonists: a French nationwide propensity-matched cohort study. Circulation 132:1252–1260

32. Nishtala PS, Gnjidic D, Jamieson HA, Hanger HC, Kaluarachchi C, Hilmer SN (2016) 'Real-world' haemorrhagic rates for warfarin and dabigatran using population-level data in New Zealand. Int J Cardiol 203:746–752

33. O'Brien EC, Simon DN, Thomas LE et al (2015) The ORBIT bleeding score: a simple bedside score to assess bleeding risk in atrial fibrillation. Eur Heart J 36:3258–3264

34. Ogawa S, Ikeda T, Kitazono T et al (2014) Present profiles of novel anticoagulant use in Japanese patients with atrial fibrillation: insights from the Rivaroxaban Postmarketing Surveillance Registry. J Stroke Cerebrovasc Dis 23:2520–2526

35. Seeger JD, Bykov K, Bartels DB, Huybrechts K, Zint K, Schneeweiss S (2015) Safety and effectiveness of dabigatran and warfarin in routine care of patients with atrial fibrillation. Thromb Haemost 114:1277–1289

36. Sørensen R, Gislason G, Torp-Pedersen C et al (2013) Dabigatran use in Danish atrial fibrillation patients in 2011: a nationwide study. BMJ Open 3:e002758

37. Steinberg BA, Kim S, Piccini JP et al (2013) Use and associated risks of concomitant aspirin therapy with oral anticoagulation in patients with atrial fibrillation: insights from the Outcomes Registry for Better Informed Treatment of Atrial Fibrillation (ORBIT-AF) Registry. Circulation 128:721–728

38. Tamayo S, Peacock FW, Patel M et al (2015) Characterizing major bleeding in patients with nonvalvular atrial fibrillation: a pharmacovigilance study of 27 467 patients taking rivaroxaban. Clin Cardiol 38:63–68

39. Vaughan Sarrazin MS, Jones M, Mazur A, Chrischilles E, Cram P (2014) Bleeding rates in Veterans Affairs patients with atrial fibrillation who switch from warfarin to dabigatran. Am J Med 127:1179–1185

40. Villines TC, Schnee J, Fraeman K et al (2015) A comparison of the safety and effectiveness of dabigatran and warfarin in non-valvular atrial fibrillation patients in a large healthcare system. Thromb Haemost 114:1290–1298

41. Yamaji H, Murakami T, Hina K et al (2013) Usefulness of dabigatran etexilate as periprocedural anticoagulation therapy for atrial fibrillation ablation. Clin Drug Investig 33:409–418

42. Yao X, Abraham NS, Alexander GC et al (2016) Effect of adherence to oral anticoagulants on risk of stroke and major bleeding among patients with atrial fibrillation. J Am Heart Assoc 5:e003074

43. Yap LB, Eng DT, Sivalingam L et al (2015) A comparison of dabigatran with warfarin for stroke prevention in atrial fibrillation in an Asian population. Clin Appl Thromb Hemost doi: 10.1177/1076029615584664

44. Yap LB, Rusani BI, Umadevan D et al (2014) A single centre experience of the efficacy and safety of dabigatran etexilate used for stroke prevention in atrial fibrillation. J Thromb Thrombolysis 38:39–44
